# Molecular assessment of collagen denaturation in decellularized tissues using a collagen hybridizing peptide

**DOI:** 10.1016/j.actbio.2017.01.079

**Published:** 2017-02-01

**Authors:** Jeongmin Hwang, Boi Hoa San, Neill J. Turner, Lisa J. White, Denver M. Faulk, Stephen F. Badylak, Yang Li, S. Michael Yu

**Affiliations:** a Department of Bioengineering, University of Utah, Salt Lake City, UT 84112, USA; b McGowan Institute for Regenerative Medicine, University of Pittsburgh, Pittsburgh, PA 15219, USA; c Department of Surgery, University of Pittsburgh, Pittsburgh, PA 15213, USA; d School of Pharmacy, University of Nottingham, Nottingham NG7 2RD, UK; e Department of Bioengineering, University of Pittsburgh, Pittsburgh, PA 15219, USA; f Department of Pharmaceutics and Pharmaceutical Chemistry, University of Utah, Salt Lake City, UT 84112, USA

**Keywords:** Extracellular matrix, Triple helix, Biologic scaffolds, Tissue engineering, Detergent

## Abstract

Decellularized extracellular matrix (ECM) derived from tissues and organs are emerging as important scaffold materials for regenerative medicine. Many believe that preservation of the native ECM structure during decellularization is highly desirable. However, because effective techniques to assess the structural damage in ECM are lacking, the disruptive effects of a decellularization method and the impact of the associated structural damage upon the scaffold’s regenerative capacity are often debated. Using a novel collagen hybridizing peptide (CHP) that specifically binds to unfolded collagen chains, we investigated the molecular denaturation of collagen in the ECM decellularized by four commonly used cellremoving detergents: sodium dodecyl sulfate (SDS), 3-[(3-cholamidopropyl)dimethylammonio]-1-propa nesulfonate (CHAPS), sodium deoxycholate (SD), and Triton X-100. Staining of the detergent-treated porcine ligament and urinary bladder matrix with carboxyfluorescein-labeled CHP demonstrated that SDS and Triton X-100 denature the triple helical collagen molecule while CHAPS and SD do not, although second harmonic generation imaging and transmission electron microscopy (TEM) revealed that all four detergents disrupt collagen fibrils. Our findings from the CHP staining were further confirmed by the circular dichroism spectra of intact triple helical collagen molecules in CHAPS and SD solutions, and the TEM images of CHP-conjugated gold nanoparticles binding only to the SDS and Triton X-100 treated collagen fibrils. CHP is a powerful new tool for direct and reliable measurement of denatured collagen molecules in decellularized tissues. It is expected to have wide applications in the development and standardization of the tissue/organ decellularization technology.

**Statement of Significance:**

Preservation of the native ECM structure in decellularized tissues is highly desirable, since denaturation of ECM molecules (e.g., collagen) during decellularization can strongly influence the cellular response. Unfortunately, conventional techniques (SEM, SHG) are not conducive to identifying denatured collagen molecules in tissues. We demonstrate the first investigation into the *molecular* denaturation of collagen in decellularized ECM enabled by a novel Collagen Hybridizing Peptide (CHP) that specifically binds to unfolded collagen chains. We show that SDS and Triton X-100 denature collagen molecules while CHAPS and SD cannot. Such detection has been nearly impossible with other existing techniques. The CHP technique will advance our understanding about the effect of the cell-removing process on ECM, and lead to development of the decellularization technology.

## 1. Introduction

Extracellular matrix (ECM) obtained by the decellularization of tissues has become an important biomaterial for tissue engineering and regenerative medicine [1]. Dozens of products derived from decellularized tissues, commonly known as biologic scaffolds, are currently used in clinical practice [1] for applications in wound care [2,3], pericardial reconstruction [4], and heart valve replacement [5], among others. By presenting a combination of structural and biological factors, such as the three-dimensional (3D) ultrastructure, mechanical integrity and a specific ECM composition, these acellular biologic scaffolds can provide a near-native and complex microenvironment for cell growth and tissue development, which is difficult to recapitulate with synthetic materials or a single ECM component. The ECM can also be partially digested with pepsin, and reconstituted *in situ* to form a hydrogel [6,7], which retains numerous biochemical constituents found in native tissues, such as growth factors and glycosaminoglycan. When the hydrogel forms in the damaged tissue, these bioactive cues can promote a constructive host remodeling response while curbing inflammation and scarring [6]. One example is the use of decellularized myocardial matrix hydrogels as a post-myocardial infarction biomaterial therapy [8]. This new therapy has produced encouraging preclinical results [9,10] and is currently under a clinical trial [6]. In recent years, improvement in whole organ decellularization techniques has enabled 3D organ scaffolds, which preserve the native tissue architecture including vascular networks [11,12]. These decellularized scaffolds can be repopulated with selected cell types *in vitro* to regenerate functional organs. So far, a variety of organs, including heart [13], kidney [14], liver [15], lung [16,17], limb [18], and pancreas [19], have been created from decellularized whole organs, and their short-term functions have been demonstrated after transplantation *in vivo*. In light of critical shortage of organ donors, this technological breakthrough provides hopes of transplanting engineered animal organs to patients with end-stage organ failure.

The goal of tissue decellularization is to remove cells and cellular remnants while retaining the 3D ultrastructure and composition of the native ECM as much as possible. Complete removal of all cellular materials is not possible, and decellularization processes inevitably cause disruption of the matrix architecture and surface ligand landscape [1,11]. Consequently, preferred methods of decellularization vary among tissues and organs. A variety of decellularization methods have been reported so far [1], including physical disruption (e.g., freeze-thaw) [20], enzymatic treatment [21], and treatment with chemical agents, such as acids and bases [22], and various detergents [15,17,23,24]. When these methods are used appropriately, the resulting ECM scaffold can be used as a cell-guiding template that contains the bioactive cues and 3D configuration beneficial for cell infiltration and proper tissue remodeling. However, for many tissues and organs, achieving adequate decellularization can disrupt the native ECM structures dramatically. Since it is difficult to make accurate assessment of structural changes to ECM and their effects on cell-matrix interactions, selecting ideal decellularization method heavily relies on anecdotal experience. Despite an increasing number of studies in the literature that directly compare different decellularization methods [22,25–28], there are many conflicting reports about the structure-disrupting effects of a given agent even when it is used to decellularize fairly similar tissues [29,30]. These disparate results are, in large part due to the lack of convenient and accurate methods for determining the structural changes in the ECM that may occur at multiple scales during the cell-removing process.

Because the fibrillar collagen is the most predominant component of ECM [31], it has been the primary target for assessing the structural damage in decellularized tissues. Histological collagen stains such as picrosirius red, and immunohistochemistry based on anti-collagen antibodies are widely used, but they can only report the changes in collagen content after decellularization, and do not provide any information about collagen structure. Scanning electron microscopy (SEM) can reveal the higher order architecture of the collagen fibers in the decellularized ECM. Unfortunately, different decellularization methods often result in drastic differences in ECM morphology, and it is nearly impossible to compare the levels of structural disruption solely by observation of the ECM morphology under SEM [32]. Microscopic methods such as second harmonic generation (SHG) and transmission electron microscopy (TEM) have been used to detect structural damage to collagen fibers after decellularization [33,34]. The reduction of SHG signal [25], as well as the disappearance of the d-banding pattern in TEM, are indications of the loss of collagen structure. However, both methods measure collagen damage by loss of signals or features, a mechanism that is not sensitive enough to detect low levels of disruption which could be the outcome of certain decellularization agents. Most importantly, all of the existing methods can only detect the structural change of collagen at the fiber level, and there has been hardly any investigation of whether a decellularization procedure can compromise the structure of a collagen molecule, the most fundamental building block of the collagen fibers.

Here we present a new study to assess the effects of decellularization agents on the molecular structure of collagen. The study was made possible by the collagen hybridizing peptide [CHP, sequence: (GPO)_9_, O: hydroxyproline] that our group developed. The CHP is designed to have a strong propensity to fold into a triple helix, a unique super-secondary protein structure that is nearly exclusively found in collagen. A CHP strand can readily bind to unfolded collagen chains by forming a hybridized triple helix through inter-strand hydrogen bonds, in a fashion analogous to primers binding to DNA strands during PCR [35,36]. The CHP has essentially no affinity to intact collagen and other biomolecules, due to lack of any binding sites and the peptide’s neutral and hydrophilic amino acids composition [37]. Our previous studies demonstrated that the CHP can bind to collagen molecules disrupted by heat [36,37], enzymatic degradation [36], or mechanical overloading (unpublished results). In this study, we show that the carboxyfluorescein labeled CHP (designated as CF-CHP) can effectively report the level and location of molecular denaturation of collagen in tissues decellularized by commonly used detergents: sodium dodecyl sulfate (SDS) [13,16,29], 3-[(3-cholamidopropyl)d imethylammonio]-1-propanesulfonate (CHAPS) [17], sodium deoxycholate (SD) [24,38], and Triton X-100 [39,40]. Our findings demonstrate that CHP can provide molecular level information about the structural damage in decellularized ECM. It could be a useful tool for advancing our understanding of the effects of structure alternation on the performance of the acellular biologic scaffolds, and for developing new and improved tissue decellularization technologies.

## 2. Materials and methods

### 2.1. Synthesis of fluorescently labeled CHP

The CHP [sequence: GGG-(GPO)_9_, where GGG is a linker, O is hydroxyproline] and the sequence-scrambled control peptide (SSCP, GGG-PGOGPGPOPOGOGOPPGOOPGGOOPPG) were synthesized using standard solid-phase Fmoc and HBTU chemistry as described previously [36,41]. The fluorophore, 5,6-carboxyfluorescein (CF) was coupled to peptides on resin in a 24 h reaction with 6 molar equivalence of CF, 6 molar equivalence of PyAOP, and 12 molar equivalence of DIPEA. The peptides were cleaved from the resin by incubation with trifluroacetic acid (TFA)/triisopropylsilane (TIS)/water (95:2.5:2.5) for over 2 h. The peptides were purified by reverse-phase HPLC on a C18 column using a linear gradient mixture of water (0.1% TFA) and acetonitrile (0.1% TFA). The molecular weights of the purified peptides were confirmed using Bruker AutoFlexIII MALDI-ToF (Bruker Daltonics).

### 2.2. Preparation of CHP-conjugated gold nanoparticles

The CHP with an N-terminal acetylated cysteine [C-GGG-(GPO)_9_] was synthesized, purified and heated at 80 °C for 5 min followed by addition to a solution of citrate-stabilized gold nanoparticle (NP), as described previously [42]. The mixture was incubated overnight at room temperature. The unconjugated peptides were removed by repeated centrifugation (20,000 rcf) and washing of the precipitated NPs with 80 °C deionized water. The concentration of CHP-NP was determined by measurement of optical absorbance at 520 nm.

### 2.3. Decellularization of porcine urinary bladder (UBM)

Porcine urinary bladder matrix was prepared according to previous reported methods [25,43]. Briefly, the bladder was opened on one side from the neck to the dome region to form a rectangular-shaped sheet, and the tunica serosa, tunica muscularis externa, tunica submucosa, and the muscularis mucosa were removed by mechanical delamination. The remaining basement membrane and tunica propria layers of the urinary bladder constitute the urinary bladder matrix. Tissue samples were then subjected to either, 3% Triton-X 100 (Sigma-Aldrich), 8 mM CHAPS (Sigma-Aldrich), 4% sodium deoxycholate (Sigma-Aldrich), 1% SDS (Bio-Rad), or water (native control) for 24 h with physical agitation (300 rpm on an orbital shaker). Scaffolds were rinsed in deionized water for 15 min with agitation (300 rpm on orbital shaker). This rinse cycle was repeated three times. Each treatment group was then washed for 24 h in deionized water with agitation (300 rpm on orbital shaker). At the end of this wash cycle, four rounds of 15 min deionized water rinse were performed and the bladders were then washed for a final 24 h in deionized water. Lastly, scaffolds were lyophilized and sterilized *via* gamma irradiation at a dose of 2 × 10^6^ RADS.

### 2.4. Staining detergent treated tissues with CF-CHP

Healthy porcine medial cruciate ligaments were a kind gift from the Jeffrey Weiss group (University of Utah). Ligaments were frozen in optimal cutting temperature (OCT) compound and cut to 5 μm thick sections which were mounted on glass slides. Before treating with detergents, the OCT compound was removed by two rounds of 5 min rinsing in 1 × PBS. All detergents were dissolved in deionized water to the predetermined weight/volume concentrations. Tissue slides were completely immersed in detergent solutions and incubated at room temperature overnight. Slides were rinsed with deionized water twice and with 1 × PBS three times to remove leftover detergents. Because CHP can self-assemble into homotrimers in solution during storage at 4 °C leaving no driving force to hybridize with unfolded collagen chains, in order to stain denatured collagen in tissue sections, CHP needs to be dissociated to monomers by heating immediately prior to use. A solution containing 15 μM of CF-CHP in 1 × PBS were heated at 80 °C for 5 min, and immediately cooled down to room temperature in an ice/water bath. One hundred microliter of the quenched CF-CHP solution was quickly added onto each tissue section. In this way, most CHP peptides were expected to remain as active monomers during the staining process, because CHP trimerization follows a third order reaction rate and takes several hours to complete at the concentration of 15 μM [44], based on previous studies on CHP triple helix folding [45] and kinetic calculations [46]. The sections covered under CF-CHP solutions were incubated in a humidity chamber for 2 h at 4 °C. After staining, the slides were rinsed three times with 1 × PBS before mounting.

The UBM samples were flash frozen in OCT compound and sectioned. The sections were blocked with 10% goat serum in 1 × PBS for 30 min at room temperature, and treated with 15 μM of CF-CHP as described above. An anti-collagen I antibody (Abcam, ab34710, 1 mg/mL) was diluted (1:500) into the quenched CF-CHP solution before the mixture was added to the sections. The slides were incubated in a humidity chamber overnight at 4 °C, and rinsed in 1 × PBS for 5 min three times. Afterwards, the sections were stained by Alexa Fluor555 labeled donkey anti-rabbit IgG H&L (4 μg/mL, Abcam, ab150074) for 1 h at room temperature, rinsed and mounted.

### 2.5. Fluorescence microscopy

Ligament tissue sections treated with predetermined concentrations of SDS were scanned using an automated Nikon wide-field fluorescence microscope (4 × objective lens, 488 nm argon gas laser). The sections of UBM stained with CF-CHP and anti-collagen I antibody were imaged by an EVOS FL auto cell imaging system (Thermo Fisher Scientific) with a 20 × objective lens (GFP light cube for CF-CHP and RFP light cube for anti-collagen I antibody). Images within each experiment were obtained under identical microscope settings (e.g., exposure time, gain) to allow direct comparison.

### 2.6. SHG imaging of collagen fibers

The detergent-treated ligament sections were imaged by a custom Prairie View Ultima multiphoton microscope (Brucker). A single excitation wavelength at 800 nm was used to simultaneously visualize collagen fibers and CF-CHP binding in the tissue. The resulting SHG and CF-fluorescence signals were detected at 435–485 nm and 500–550 nm respectively. Images were scanned with Z-stack for the entire 5 μm section depth. Images were acquired from three different locations within one section from each treatment category. Images for both SHG and CF-fluorescence were analyzed in ImageJ software using measurements of the mean intensity and area of all remaining pixels after a background subtraction. The signal quantification was expressed as integrated intensity (the mean intensity times area). The image of the heat denatured ligament section was not included in the CF-fluorescence quantification because of over saturated intensity.

### 2.7. Circular dichroism (CD) spectroscopy

The collagen-detergent mixture solutions were freshly prepared from acid solubilized collagen to minimize potential collagen fibril self-assembly and to allow direct monitoring of the effect of a detergent on individual collagen molecules. Rat tail, type I collagen (0.3 mg/mL, Corning, Cat# 354236) in blank and four different detergent solutions were freshly prepared immediately prior to the CD measurement (no detergent: 0.3 mg/mL of collagen in a solution of 20 mM acetic acid; SDS: 0.3 mg/mL of collagen in a solution of 20 mM acetic acid containing 1% SDS; CHAPS: 0.3 mg/mL of collagen in a solution of 20 mMacetic acid containing 8 mM CHAPS; SD: 0.3 mg/mL of collagen in a 1 × PBS buffer solution containing 4% SD). CD spectra (200–260 nm) were recorded on a JASCO J-1500 CD spectrometer at 22 °C with a scanning speed of 20 nm/min, a data integration time of 16 s, and a bandwidth of 5 nm. After heating all solutions up to 65 °C, the spectra of the denatured samples were measured again at 22 °C.

Proteins in detergent solutions can aggregate and interfere with CD measurements. In this experiment, the combinations of detergent and buffer shown above were carefully chosen for minimal background CD signals. Unfortunately, we were unable to find a condition that provides a noise-free CD spectrum for collagen solutions containing 3% Triton X-100.

### 2.8. Transmission electron microscopy

A neutralized solution containing 0.6 mg/mL of rat tail, type I collagen was prepared by diluting the acidic stock solution (Corning, Cat# 354236, 4.89 mg/mL) in 1 × PBS. The neutralized collagen solution was incubated at room temperature under magnetic stirring for 2 h to allow collagen fibril self-assembly. Subsequently, the neutralized collagen solution was aliquoted into four vials, and 1 × PBS buffer solutions containing four different detergents at 2 × concentrations were added into individual vials to make four final solutions (0.3 mg/mL of collagen with 1% SDS, 8 mM CHAPS, 4% SD, or 3% Triton X-100). The collagen fibrils were incubated with detergents under magnetic stirring at room temperature for 3 h to allow detergent-fibril interaction. The collagen fibrils assembled in the detergent-free PBS solution were denatured in 80 °C water bath for 1 min as a control sample. TEM samples were prepared by adding a drop of a collagen-detergent mixture solution onto a copper grid covered with a thin carbon film (EMS). The excess solution was wicked off with a piece of blotting paper. The samples were air dried at room temperature. To visualize unfolded collagen molecules under TEM, a drop of CHP-NP (10 nM) solution containing 2% bovine serum albumin was applied to the grid. After 10 min of incubation at room temperature, the grid was washed with deionized water three times. Finally, the samples were stained with a drop of 2% uranyl acetate (w/v) and air-dried at room temperature. TEM images were collected on a FEI Tecnai T12 microscope operated at 120 kV, and processed by a Gatan digital micrograph software. The collagen-bound nan-oparticles in the TEM images were carefully identified and manually marked as red dots using the overlay brush tool in ImageJ.

### 2.9. Statistical analysis

Unless indicated, all experiments were repeated three times. All quantitative results are shown as means + standard error. All statistical analyses were performed using Kaleidagraph 4.0 (Synergy Software). Sample groups were compared using *t*-tests. When there were more than one pair of comparison in an experiment, the *t*-tests were performed with the Benjamini-Hochberg correction for multiple comparisons with a significance (false discovery rate) of 0.05 [47].

## 3. Results

### 3.1. CF-CHP mediated detection of denatured collagen molecules in detergent treated tissues

**Fig. 1:**
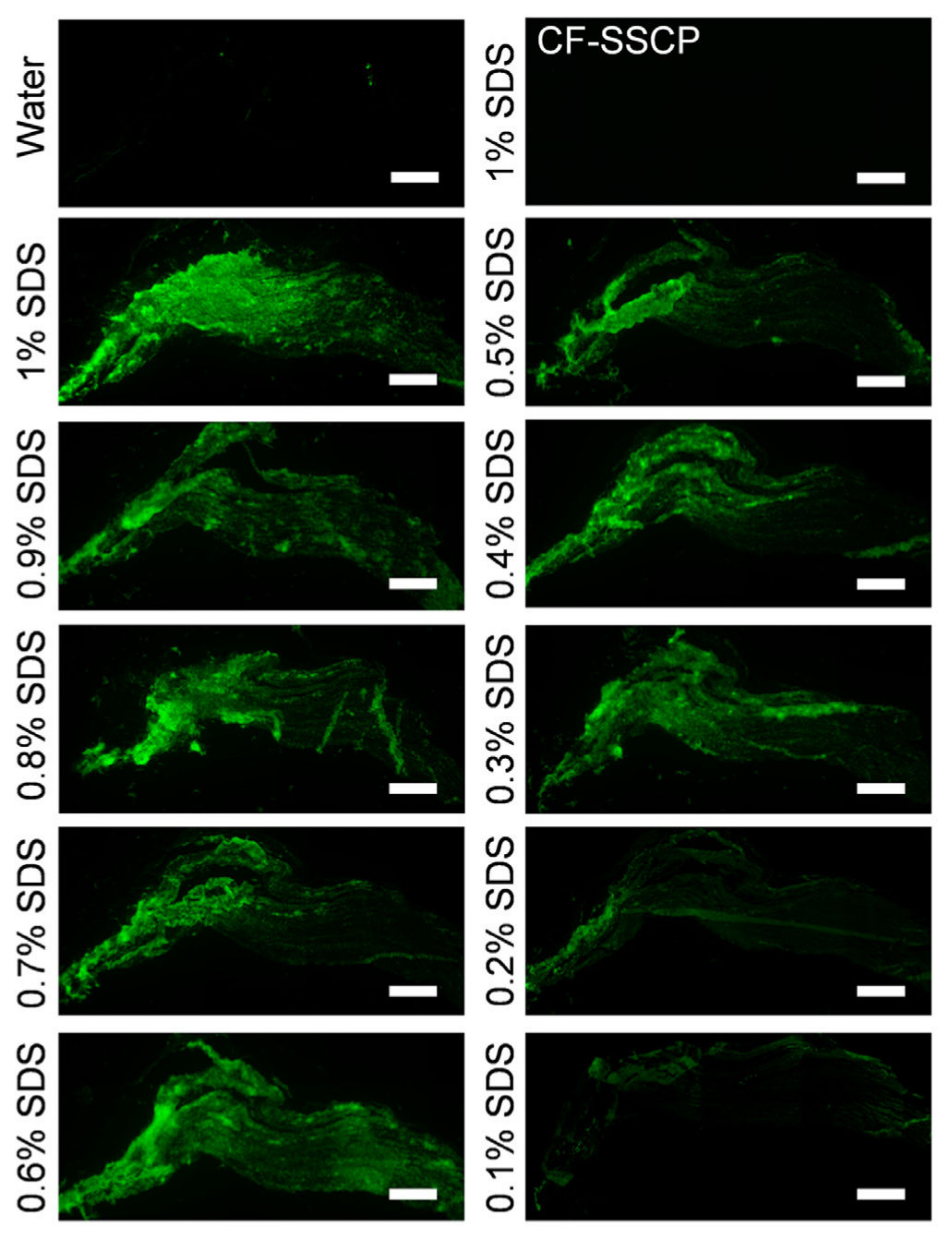
Fluorescence images showing CF-CHP staining on sections of porcine ligament treated by deionized water or SDS solutions of varying concentrations. The image of the 1% SDS treated section stained by the sequence-scrambled control peptide CF-SSCP is shown on the top right corner. Scale bar: 1 mm.

SDS is a well-known protein denaturant commonly used in electrophoresis. It is also one of the most widely utilized detergents for tissue decellularization, often in the concentration range of 0.1% to 1% (w/v), and its disrupting effect on the ECM has been recognized [1,43]. We envisioned that the collagen molecules in the tissue could be denatured by SDS treatment, exposing unfolded collagen chains which can be detected by CHP hybridization. We selected ligament as the model tissue to verify this hypothesis, not only because it consists primarily of collagen, but also because the ligament tissue was reported to become more susceptible to trypsin digestion after SDS treatment [29], which implied possible structural alteration to collagen. Glass slide-mounted neighboring cryosections of a porcine ligament were incubated overnight in either pure water or a serial dilution of SDS solutions with concentrations ranging from 0.1% to 1% at room temperature, and washed extensively followed by staining with CF-CHP. We chose to compare sections from a single tissue sample to avoid possible variation in tissue integrity resulting from multiple samples. As expected, the section treated with 1% SDS showed strong fluorescence signals from CF-CHP staining, whereas no fluorescence intensity was detected from the control section incubated in pure water (Fig. 1). It was found that complete removal of SDS from tissues after decellularization is difficult to achieve [11,43]. To ensure that the CF-CHP is binding to denatured collagen and not nonspecifically to residual SDS in the tissues, we examined another 1% SDS treated tissue section using a CF-labeled sequence-scrambled control peptide (CF-SSCP). This control peptide is identical to CHP in amino acid composition but cannot form the collagen triple helix due to the randomized sequence. The CF-SSCP showed no affinity to the SDS-treated tissue (Fig. 1 top right corner), confirming that the binding of CF-CHP was truly due to the triple helical hybridization between CHP and the collagen molecules unfolded by the SDS treatment. In addition, strong CF-CHP fluorescence intensities were observed from sections treated with SDS as dilute as 0.3%, while the 0.1% sample showed almost no signal except at the edges of the tissue section (Fig. 1). These results demonstrated that the denaturation effect of SDS on collagen molecules is attenuated at lower concentrations, and that the different levels of collagen denaturation in detergent treated tissues can be distinguished by CHP staining.

**Fig. 2:**
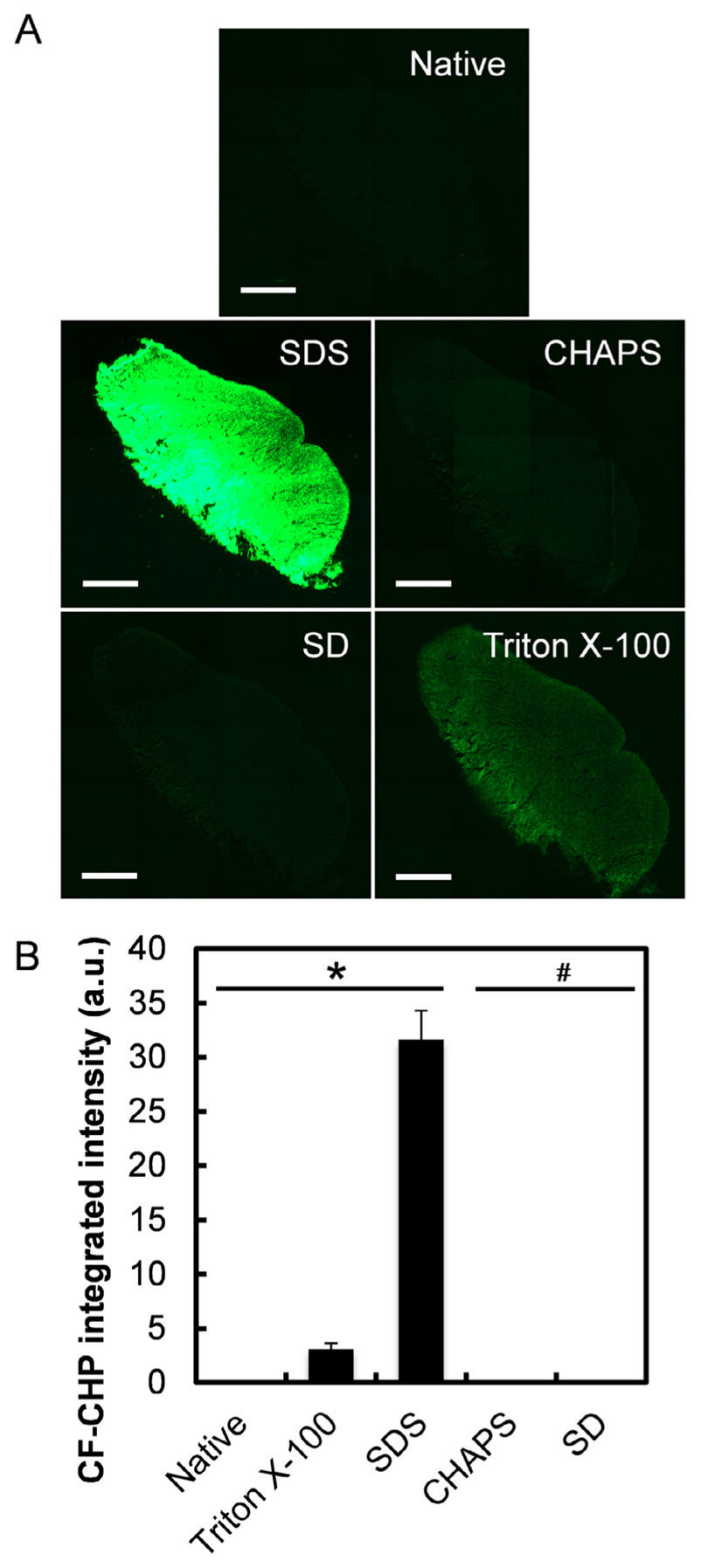
(A) Representative fluorescence images showing CF-CHP staining on porcine ligament sections treated with water (native) or different detergents (1% SDS, 8 mM CHAPS, 4% SD, 3% Triton X-100). Scale bar: 2 mm. (B) Digital quantification of the CF-CHP fluorescence intensities, arbitrary units (a. u.). Numbers are presented as mean + standard error (n = 3 for native, CHAPS, and SD; n = 4 for SDS and Triton X-100). Pairs of means under the horizontal line marked with * are significantly different from each other; the two groups under the line marked with # are not significantly different from the native control.

We selected four detergents (1% SDS, 3% Triton X-100, 4% SD, and 8 mM CHAPS, chemical structures shown in Fig. S1) with representative concentrations (w/v%) because of their frequent use in the decellularization process [1,13,15,17,25,48,49]. Neighboring cryosections of a ligament tissue were subjected to different detergent treatments overnight and were subsequently stained by CFCHP. Whole section fluorescence scans revealed apparent CF-CHP signals in 1% SDS and 3% Triton X-100 treated sections, indicating presence of denatured collagen molecules. Visual observation (Fig. 2A) and digital quantification of the fluorescence signals in the scanned images (Fig. 2B) both showed that the SDS treated samples contain over an order of magnitude more denatured collagen molecules than the Triton X-100 treated samples. Sections treated with pure water (native tissue), 8 mM CHAPS and 4% SD did not show recognizable CF-CHP intensity (Fig. 2A & B), suggesting that CHAPS and SD do not cause significant unfolding of collagen molecules. CF-CHP was also used to stain another set of tissue sections which were thoroughly heated in 90 °C water bath (Fig. S2). Assuming all collagen molecules were denatured in the heated sample, the average CF-CHP signals quantified from the 1% SDS treated samples correspond to approximately 11.9% of the total collagen content in the tissue section (Fig. S2B). The CFCHP staining also revealed that collagen denaturation by SDS and Triton X-100 seemed to concentrate in certain areas, whereas heat induced denaturation resulted in a global denaturation of collagen (Fig. S2A). In addition, we found that CHAPS and SD were unable to denature collagen even when their concentrations were doubled (16 mM CHAPS and 8% SD), and that the level of denaturation was reduced when the concentration of Triton X-100 was lowered (Fig. S3).

### 3.2. Dual channel imaging of SHG and CF-CHP fluorescence

**Fig. 3:**
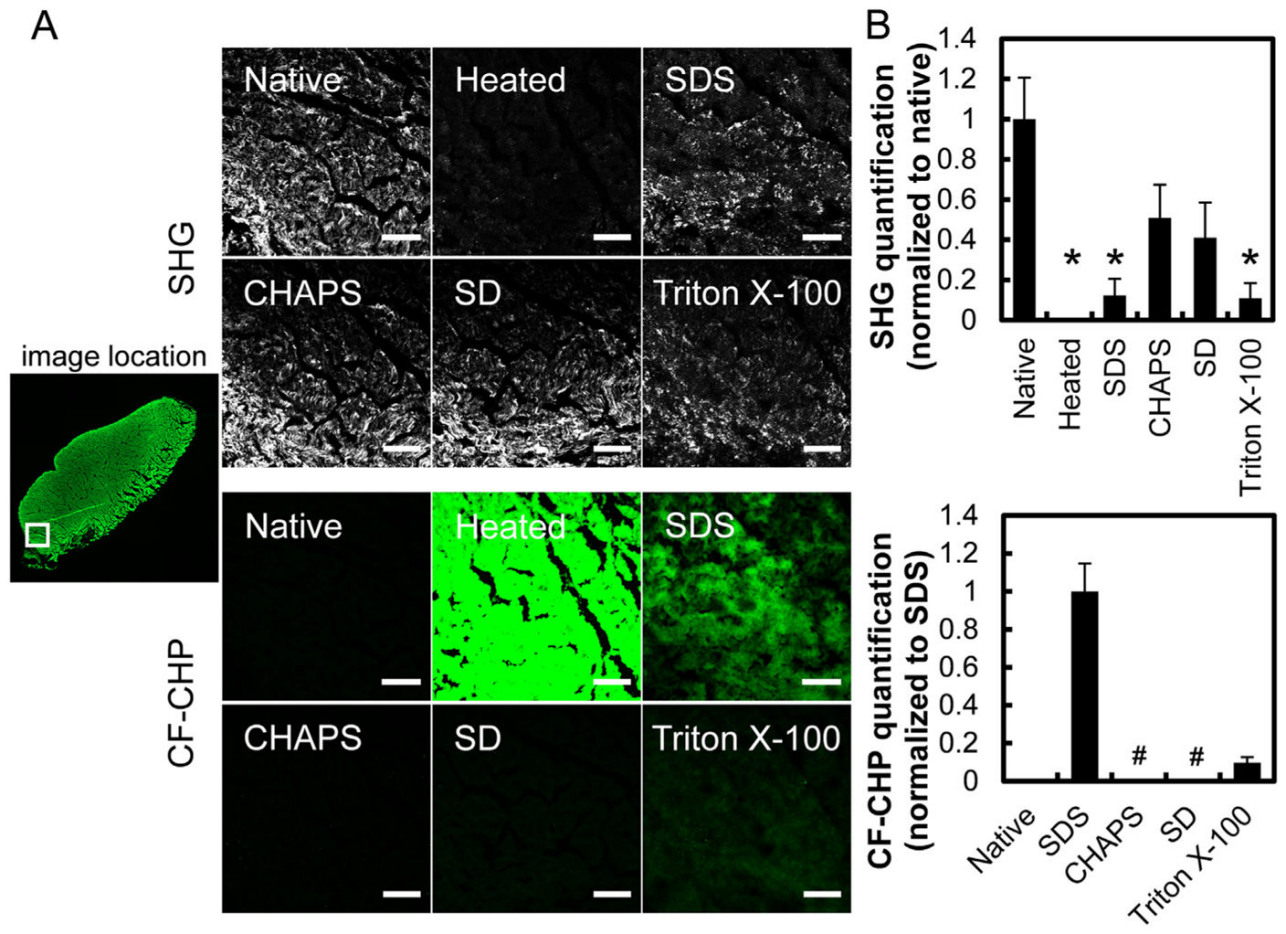
(A) Representative views from simultaneous multiphoton imaging of native or heat denatured porcine ligament tissue section, or sections treated with detergents (1% SDS, 8 mM CHAPS, 4% SD, 3% Triton X-100) showing SHG signals from intact collagen fibers and fluorescence intensities from CF-CHP binding to denatured collagen. All images were taken from the same location of the tissue sections (marked by the white box in the whole section scan image on the left). Scale bar: 150 μm. (B) The integrated SHG signals (normalized to the intact native sample) and CF-CHP signals (normalized to the 1% SDS treated sample) quantified from images of three random locations within each tissue section. Images from the other two locations are shown in Figs. S4 and S5. Numbers are presented as mean + standard error. The * sign indicates statistical significance in comparison to the native control group; the groups marked by # are not significantly different from the native group.

To corroborate our findings, we conducted second harmonic generation imaging, a classic technique that can interrogate the collagen fiber integrity, in conjunction with the fluorescence microscopy. The ligament samples were imaged using multiphoton confocal microscopy under a single excitation wavelength, acquiring the collagen fiber’s SHG signal on one channel, and the CF-CHP’s two-photon excitation fluorescence (TPEF) signal on another channel simultaneously. Comparing the stacked SHG micrographs and their quantified signals acquired from the same location within each tissue section (Fig. 3A), we were able to determine that the strong SHG signal from the orderly packed collagen fibers present in the native tissue was completely absent in the heated tissue and greatly diminished in the SDS and Triton X-100 treated samples. Similar results were also found for images from two other locations (Figs. S4 and S5) and confirmed by the quantified signals (Fig. 3B). The diminished SHG results strongly indicate disruption of the collagen structure by SDS and Triton X-100, which also correlate with the concurrently acquired positive CF-CHP signals that are indicative of structural damages to collagen (Fig. 3A bottom). In addition, the images of CF-CHP stain in the SDS-denatured ligament tissue appeared in a diffusive and amorphous form lacking the distinct shape of collagen fibers (Figs. 3A, S4 and S5). This morphology suggests disruption of the collagen fiber structure by SDS, correlating with the diminished SHG signals. The SHG and CHP data confirmed the disruption of collagen structure by 1% SDS and 3% Triton X-100, at both the fibrillar and molecular levels. On the other hand, the SHG signals from the tissue sections treated with 8 mM CHAPS or 4% SD often seemed to be lower than the native control, whereas no fluorescence intensity was detected after CF-CHP staining (Figs. 3A and B, S4 and S5), suggesting that the two detergents may only disrupt the *fibrillar organization* of collagen in the tissue while leaving the collagen molecules intact. This result was unexpected, since previous studies using SEM and SHG indicated that CHAPS and SD clearly disrupt collagen structure in the decellularized tissues [25]. To confirm our findings, we further characterized the collagen structure at both the molecular and fibrillar level using CD and TEM.

### 3.3. CD study of collagen triple helix in detergent solutions

**Fig. 4:**
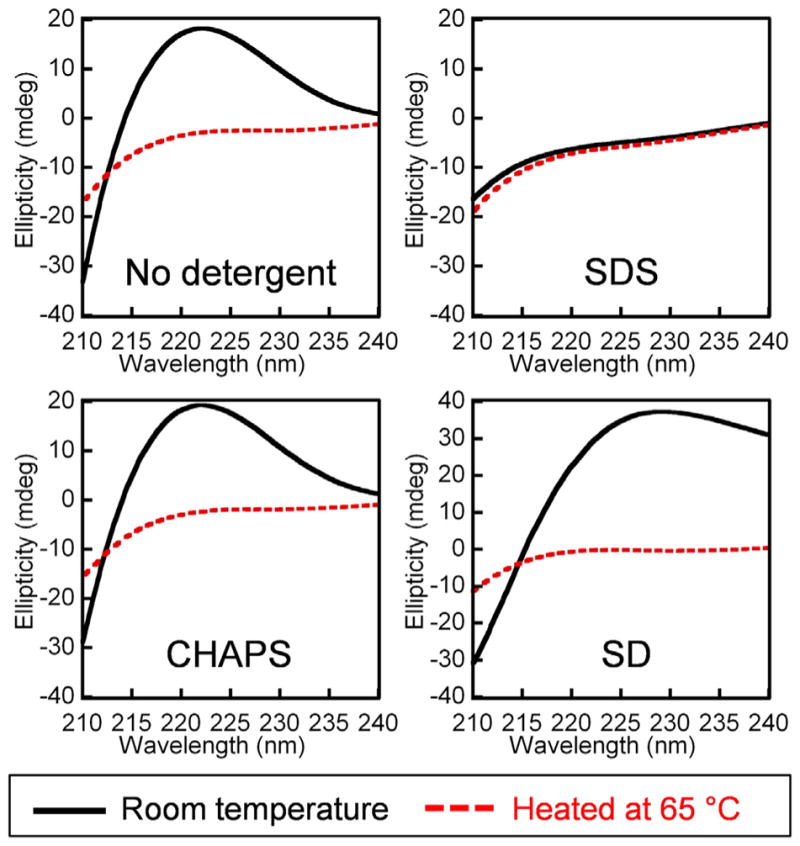
CD spectra of soluble rat tail type I collagen in solutions with or without detergents (1% SDS, 8 mM CHAPS, 4% SD). The spectra in black were recorded with freshly prepared samples at room temperature. The spectra in red were recorded after heating each sample at 65 °C.

To verify the intact structure of collagen molecules in 8 mM CHAPS and 4% SD, we acquired the CD spectra of collagen molecules in the detergent solutions (Fig. 4), since CD can reliably report the conformational changes of the collagen triple helix in solution [41,50]. Except 3% Triton X-100, we managed to prepare stable solutions containing collagen and each detergent without significant protein aggregation and light scattering, allowing acquisition of reliable CD spectra (see Methods). Without detergents, the triple helical structure of intact collagen produced to a characteristic peak near 220 nm, which disappeared when the protein was denatured after heating. The CD spectrum of collagen in 1% SDS showed no peak near 220 nm, and it overlapped with the spectrum of heat-denatured collagen almost completely, suggesting that the triple helix is unfolded by SDS. In contrast, samples in 8 mM CHAPS and 4% SD exhibited a strong positive peak near 220 nm, indicating intact triple helix (Fig. 4). These CD data agree with the CF-CHP staining results and demonstrate that unlike SDS, the CHAPS and SD lack the capacity to denature the collagen triple helix.

### 3.4. TEM study of detergent-treated collagen fibrils

**Fig. 5:**
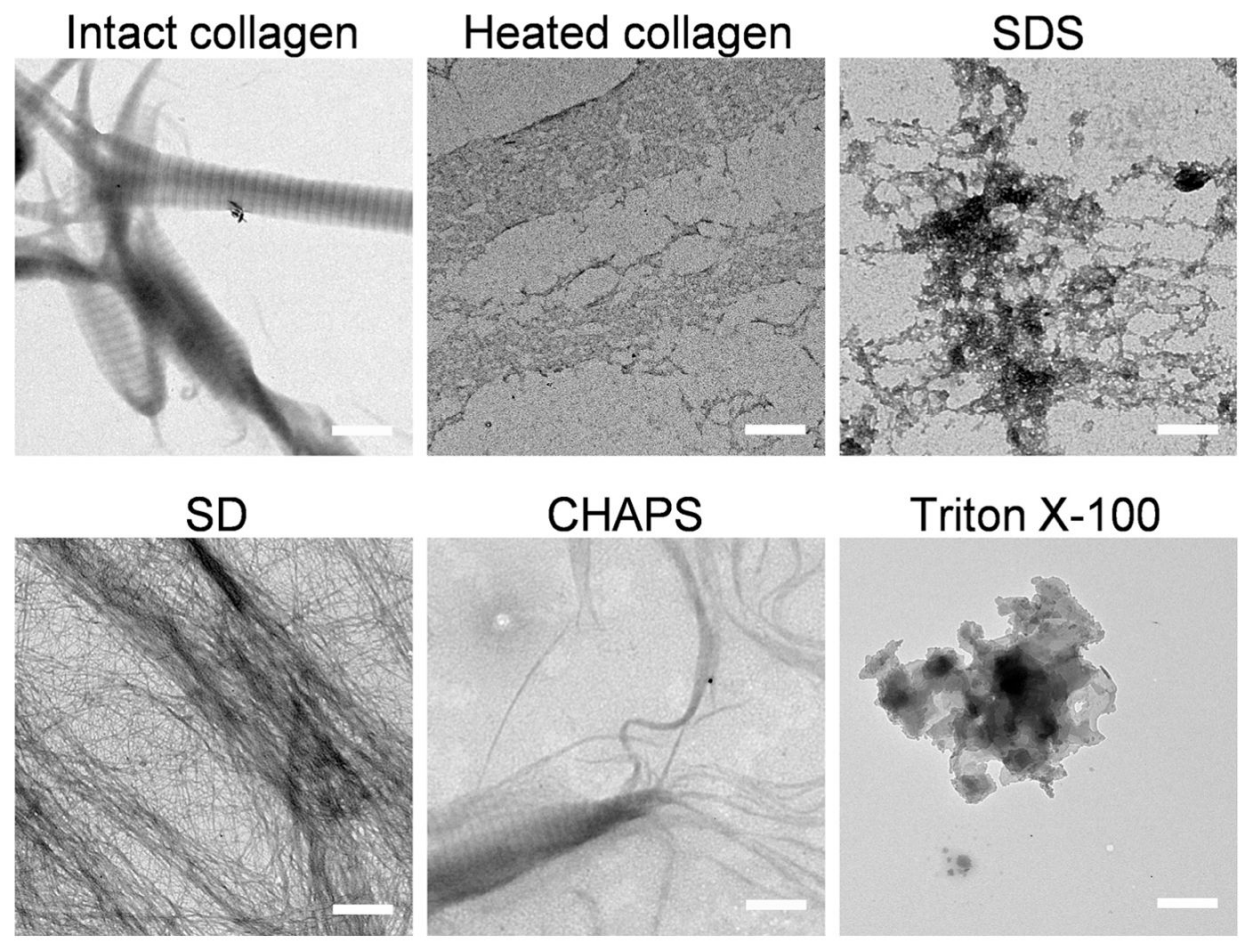
TEM images of reconstituted and self-assembled rat tail type I collagen fibrils in detergent-containing PBS solutions (1% SDS, 8 mM CHAPS, 4% SD, 3% Triton X-100). Intact fibrils in blank PBS before and after heat denaturation are shown as controls. Scale bar: 500 nm.

To verify the disruption to the fibrillar structure, we analyzed the morphology of the collagen fibrils (reconstituted and assembled from acid-soluble type I collagen) after they were mixed with each of the four detergents (Fig. 5). The representative TEM images showed that the characteristic d-banding pattern of intact type I collagen fibril was completely destroyed when the fibrils were heated or incubated with 1% SDS, where only randomly-shaped protein aggregates could be found. The fibril morphology was also largely disrupted into unstructured smears by Triton X-100 (Fig. 5). In the 8 mM CHAPS and 4% SD solutions, the regular collagen fiber bundles seemed to disassemble into smaller fibrils whose widths were on the order of tens of nanometers. Such dissociation of large fiber into smaller fibrils could be clearly seen in the CHAPS sample (Fig. 5). The presence of the smaller but well-defined fibril structures in the CHAPS and SD samples implies that the basic structural unit of the protein assembly is intact.

**Fig. 6:**
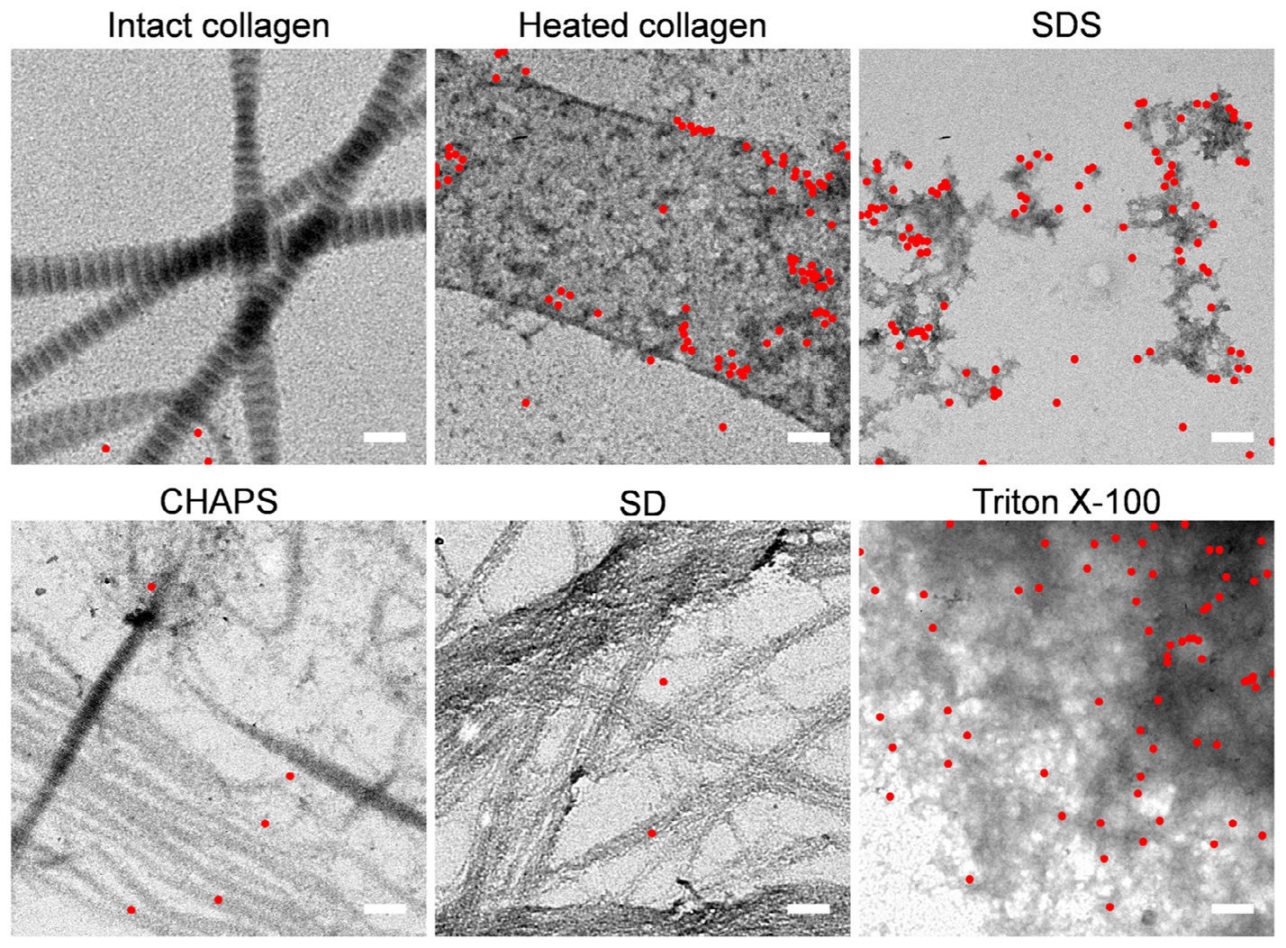
TEM images of 10 nm gold nanoparticles conjugated with CHP (CHP-NPs) binding on reconstituted rat tail type I collagen fibrils treated with detergents (1% SDS, 8 mM CHAPS, 4% SD, 3% Triton X-100). Intact fibrils in blank PBS before and after heat denaturation are shown as controls. CHP-NPs are marked with red dots for visual assistance. More representative raw images can be found in supplementary data. Scale bar: 200 nm.

To verify whether these detergent-modified collagen structures contain denatured collagen molecules, we probed the TEM samples with gold nanoparticles that have CHPs on their surface (designed as CHP-NP). Previously using TEM, we demonstrated that CHP-NPs bind to both thermally denatured [42] and mechanically damaged collagen molecules (unpublished results) by triple-helical hybridization. Here we found high densities of collagen-bound CHP-NPs in the samples subjected to heating, 1% SDS, and 3% Triton X-100, whereas only a few NPs could be seen in the native collagen sample, as well as in samples subject to 8 mM CHAPS and 4% SD (Fig. 6). Additional TEM images are in the Supplementary data (Figs. S6–11). These images provide the most direct evidence that CHAPS and SD only alter the fibrillar structures of type I collagen and do not denature the protein molecule, while SDS and Triton X-100 can disrupt the collagen structures at both molecular and fibrillar levels.

### 3.5. Examination of decellularized urinary bladder matrix using CHP

**Fig. 7:**
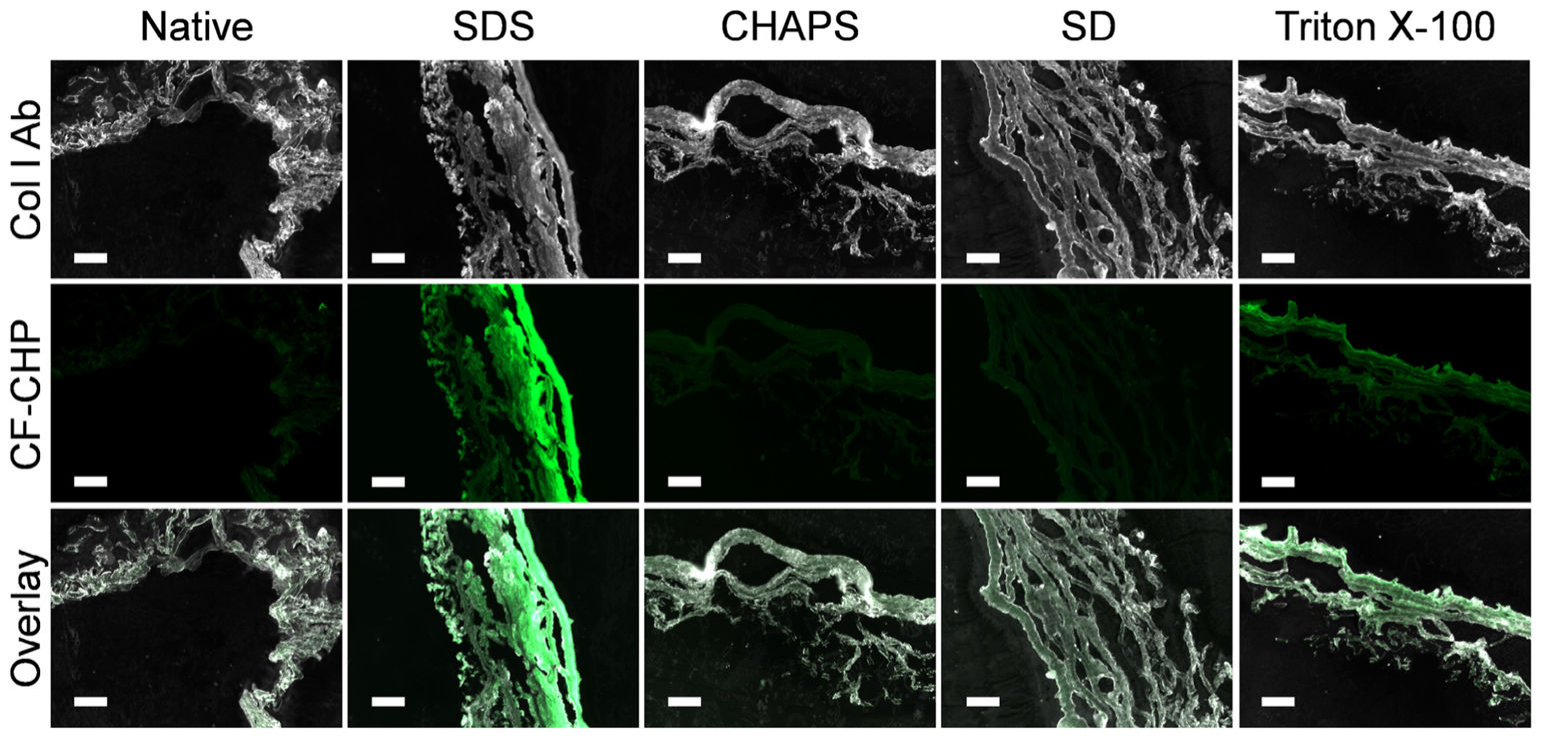
Micrographs of sections of porcine urinary bladder matrices decellularized with different detergents (1% SDS, 8 mM CHAPS, 4% SD, 3% Triton X-100) stained by CF-CHP (green) and anti-collagen I antibody (white), in comparison to the native tissue. Scale bar: 50 μm.

Finally, we conducted a field test of staining detergent treated UBM samples with CF-CHP in order to detect denatured collagen. Four pieces of tissues from the same porcine urinary bladder were decellularized by each of the four detergents (see Methods), cryosectioned, and stained by the CF-CHP. The levels of cell removal by the four detergents have been evaluated by dsDNA quantification and mass spectroscopy in the previous studies [25,43]. Here, the fluorescence images from the stained matrices (Figs. 7 and S12) clearly showed that both 1% SDS and 3% Triton X-100 caused denaturation of collagen molecules in the UBM, with 1% SDS having a much stronger denaturing effect, whereas 8 mM CHAPS and 4% SD produced negligible denaturation. These results are in strong agreement with the data obtained from the ligament tissue (Figs. 2 and 3) and the self-assembled collagen fibrils (Figs. 5 and 6).

## 4. Discussion

In this study, CHP was used to investigate the denaturation of triple helical collagen molecules in ECM-based tissue scaffolds after they were decellularized by common detergent treatment protocols. The reduced SHG signals (Fig. 3) and non-native fibril morphology displayed under TEM (Fig. 5) demonstrated that all four detergents (SDS, CHAPS, SD, and Triton X-100) can alter the structure of collagen fibrils. Moreover, the CHP staining (Figs. 1, 2, and 7), CD spectra (Fig. 4), and TEM images of CHP-NP binding (Fig. 6) showed that SDS and Triton X-100 are able to unravel the triple helix of a collagen molecule, while CHAPS and SD are unable to do so, even when their concentrations are doubled from common decellularization conditions (Fig. S3). The results were consistent in all three forms of collagen tested: solubilized type I collagen molecules, reconstituted and self-assembled type I collagen fibrils, and native collagen fibers in two different types of tissue (porcine ligament and UBM). We recently reported an in depth study of the effects of detergents on the structure and composition of the UBMECM, using immunohistochemistry, SEM and SHG imaging [25]. In the report, the multiphoton images of the CHAPS- and SDS-treated matrices showed similar levels of reduction of SHG signals, and their SEM images both showed amorphous structures lacking distinct fibers [25], suggesting that the two detergents had similar collagen denaturing properties. In contrast, the CHP hybridization study (Fig. 7) revealed that the two detergents have very different denaturing effects at the molecular level: the UBM matrix decellularized by 1% SDS contains substantial amounts of denatured collagen, whereas the UBM decellularized by CHAPS contained almost no denatured collagen. Such results are now self-evident because the denatured collagen molecules in a decellularized tissue can be directly visualized by a specific positive signal.

The protein-denaturing power of detergents depends on a combination of structural factors such as charge density of hydrophilic groups, size and rigidity of hydrophobic groups, as well as specific interactions between detergents and proteins [51–53]. The denaturation of collagen molecules in the ECM does not seem to be directly related to the charge status of the detergents: SDS and SD are anionic; CHAPS is zwitterionic; Triton X-100 is non-ionic. Interestingly, CHAPS and SD which do not denature collagen molecules contain a rigid and bulky steroid moiety, while the collagen-denaturing SDS and Triton X-100 have linear and relatively flexible structures (Fig. S1). These findings are in agreement with prior reports that detergents with rigid hydrophobic groups are less damaging to membrane proteins. In fact, there are abundant studies and extensive insights about detergent-protein interactions in the field of membrane protein biophysics [51–53], since they are often isolated in biologically active forms using detergent solutions. We believe that existing knowledge in the membrane protein community can give guidance in selection or even creation of new detergents ideal for decellularized biologic scaffolds [54].

Cells can readily recognize denaturation of the collagen triple helix. Many cell receptors, such as DDR1, DDR2, and integrins α2β1, α1β1, α10β1, α11β1, only bind to sites on triple helical collagen and not the denatured collagen chains [55]. These receptors regulate fundamental cellular processes including proliferation, adhesion, and migration. On the other hand, denatured collagen exposes cryptic cell binding sites that are inactive in the triple helical conformation, such as multiple RGD sequences recognized by another subset of integrins [56]. Therefore, unraveling of the collagen triple helix will lead to changes in behavior of cells in the ECM. In addition, it is known that peptide fragments from *in vivo* digestion of the decellularized ECM have potent bioactivities and contribute to the tissue repair processes [54]. The denatured collagens is prone to enzymatic digestion which could result in fast release of these bioactive peptide fragments. The overall effect of the denatured collagen in the scaffolds on tissue regeneration, whether favorable or adverse, may depend on the specific tissue type and the denaturation level, and its full understanding will require further investigation. We believe that CHP will be one of the most useful tools for such investigation.

Our study provides an opportunity to reconcile the conflicting literature reports about the denaturing effect of common decellularization agents. For example, a study by Gratzer and coworkers showed that 1% SDS disrupted collagen in porcine anterior cruciate ligament and altered its mechanical strength [29], while another study by Dunn and coworkers showed that the same 1% SDS solution did not have apparent effect on collagen in rat tail tendons [30], a tissue that is fairly similar to ligament in composition and structure. Our investigation clearly suggests that 1% SDS denatures collagen molecules in tendon and ligament. In Dunn’s report, the collagen denaturation was evaluated by comparing the mechanical strengths of native and detergent-treated tendons after incubation with trypsin at 37 °C [30]. Since the intact triple helical collagen is resistant to trypsin digestion, the decreased mechanical strength of the tissue after trypsin treatment was considered an indication of collagen denaturation [30]. Because the authors found that the decrease of mechanical strength of the SDS-treated samples after trypsin incubation was not greater than that of the native tissue, they concluded that decellularization by 1% SDS retains near normal structure of the collagenous tissue [30]. However, it is known that trypsin can remove the non-triple-helical telopeptides [57] and solubilize collagen from tissues [58], and even directly cleave the collagen triple helix at 37 °C, probably through the locally unfolded thermal labile domains [59]. Because of these factors, the mechanical strength of even the native tendon will be significantly reduced after trypsin treatment, largely obscuring the denaturing effect of SDS. Indeed, their assay showed that even the native tissue exhibited 34.6% decrease in mechanical strength after trypsin incubation, a ratio that is too high to be solely accounted by the digestion of the inherent trace amount of denatured collagen in tendon (approximately 0.2%–2.5% [60]). Based on the results of the present study, we think that the 1% SDS treatment did result in collagen denaturation in Dunn’s study, but this was not noticed in their mechanical test because the background value from the control group was too high. We also think that the detergents’ impact on the molecular structure of collagen does not depend on specific tissue types, and that it can be mitigated by lower detergent concentrations (Fig. S3) or shorter treatment time, although such mild conditions may not be conducive to efficient cell removal.

CHP staining is a reliable and highly sensitive method to examine the unfolding of collagen molecules in decellularized ECM, and it has many advantages over conventional methods. The mechanical or trypsin digestion method involves too many error-prone steps that undermine the detection sensitivity and accuracy as can be seen in the above-mentioned example. Microscopic methods such as SEM and TEM are not quantitative. Moreover, as demonstrated in our previous SEM work on gelatin, type I collagen, and their mixtures, highly denatured collagen (gelatin) are invisible on SEM [61]. In contrast, CHP can readily visualize denatured collagen, and with simple image analysis, it allows quantitative estimation of the level of denaturation. Analyzing large tissues using TEM and SHG imaging can be time consuming and cost prohibitive, but it is fast and simple to do so with CHP staining. Only four detergents were tested in this study as a proof of concept, but CHP staining can be readily used to study other decellularization methods based on enzymes, freeze thawing, hypotonic solutions, and pressure. We envision that CHP could facilitate researchers and manufacturers of ECM-based medical products to evaluate and optimize not only decellularization procedures but also sterilization protocols (e.g., gamma irradiation, ethylene oxide gas) [62]. Also, because CHP can bind to degraded collagen in degenerative and pathological conditions [36], it could be used prior to decellularization of tissues or organs to examine the presence of any age or disease related structural damage in the ECM which could negatively affect the scaffold’s performance.

## 5. Conclusion

We characterized the molecular denaturation of collagen in tissues as a result of common detergent treatment (SDS, CHAPS, SD and Triton X-100). The study was made possible by a novel collagen hybridizing peptide that specifically anneals to the unfolded collagen chains through triple helix formation. By staining tissue samples with fluorescently labeled CHP and subsequent quantitative image analysis, we showed that while all four detergents alter the fibrillar structure of collagen, only SDS and Triton X-100 have the propensity to denature the collagen molecules. The results were consistent in all test samples: self-assembled type I collagen fibrils, decellularized porcine ligament, and urinary bladder matrix. The results were also in agreement with other characterization methods, including SHG, CD and TEM. The triple helix is the most fundamental structural motif of collagen, and its integrity, which is known to affect cellular responses, is directly assessed in the decellularized ECM in our study. There are growing clinical interests in the use of decellularized biologic scaffolds for healing and regeneration of damaged tissues. The CHP technique introduced herein can help identify one of the key variables in the decellularization process — the molecular level destruction of collagen, and is expected to bring us one step closer to the understanding of how the decellularization process results in structural changes in the ECM and how that change influence the functional outcome of the ECM scaffolds. These new insights will ultimately lead to rationally optimized tissue decellularization protocols for specific regenerative applications.

## Appendix A. Supplementary data

Supplementary data associated with this article can be found, in the online version, at http://dx.doi.org/10.1016/j.actbio.2017.01.079.

## References

[1] Crapo PM, Gilbert TW, Badylak SF (2011). An overview of tissue and whole organ decellularization processes. Biomaterials.

[2] Mostow EN, Haraway GD, Dalsing M, Hodde JP, King D, OASIS Venus Ulcer Study Group (2005). Effectiveness of an extracellular matrix graft (OASIS Wound Matrix) in the treatment of chronic leg ulcers: a randomized clinical trial. J Vasc Surg.

[3] Cornwell KG, Landsman A, James KS (2009). Extracellular matrix biomaterials for soft tissue repair. Clin Podiatric Med Surg.

[4] Boyd WD, Johnson WE, Sultan PK, Deering TF, Matheny RG (2010). Pericardial reconstruction using an extracellular matrix implant correlates with reduced risk of postoperative atrial fibrillation in coronary artery bypass surgery patients. Heart Surg Forum.

[5] Dasi LP, Simon HA, Sucosky P, Yoganathan AP (2009). Fluid mechanics of artificial heart valves. Clin Exp Pharmacol Physiol.

[6] Wang RM, Christman KL (2016). Decellularized myocardial matrix hydrogels: in basic research and preclinical studies. Adv Drug Del Rev.

[7] Wolf MT, Daly KA, Brennan-Pierce EP, Johnson SA, Carruthers CA, D’Amore A, Nagarkar SP, Velankar SS, Badylak SF (2012). A hydrogel derived from decellularized dermal extracellular matrix. Biomaterials.

[8] Singelyn JM, DeQuach JA, Seif-Naraghi SB, Littlefield RB, Schup-Magoffin PJ, Christman KL (2009). Naturally derived myocardial matrix as an injectable scaffold for cardiac tissue engineering. Biomaterials.

[9] Seif-Naraghi SB, Singelyn JM, Salvatore MA, Osborn KG, Wang JJ, Sampat U, Kwan OL, Strachan GM, Wong J, Schup-Magoffin PJ, Braden RL, Bartels K, DeQuach JA, Preul M, Kinsey AM, DeMaria AN, Dib N, Christman KL (2013). Safety and efficacy of an injectable extracellular matrix hydrogel for treating myocardial infarction. Sci Transl Med.

[10] Singelyn JM, Sundaramurthy P, Johnson TD, Schup-Magoffin PJ, Hu DP, Faulk DM, Wang J, Mayle KM, Bartels K, Salvatore M, Kinsey AM, DeMaria AN, Dib N, Christman KL (2012). Catheter-deliverable hydrogel derived from decellularized ventricular extracellular matrix increases endogenous cardiomyocytes and preserves cardiac function post-myocardial infarction. J Am Coll Cardiol.

[11] Guyette JP, Gilpin SE, Charest JM, Tapias LF, Ren X, Ott HC (2014). Perfusion decellularization of whole organs. Nat Protoc.

[12] Peloso A, Dhal A, Zambon JP, Li P, Orlando G, Atala A, Soker S (2015). Current achievements and future perspectives in whole-organ bioengineering. Stem Cell Res Ther.

[13] Ott HC, Matthiesen TS, Goh SK, Black LD, Kren SM, Netoff TI, Taylor DA (2008). Perfusion-decellularized matrix: using nature’s platform to engineer a bioartificial heart. Nat Med.

[14] Song JJ, Guyette JP, Gilpin SE, Gonzalez G, Vacanti JP, Ott HC (2013). Regeneration and experimental orthotopic transplantation of a bioengineered kidney. Nat Med.

[15] Uygun BE, Soto-Gutierrez A, Yagi H, Izamis ML, Guzzardi MA, Shulman C, Milwid J, Kobayashi N, Tilles A, Berthiaume F, Hertl M, Nahmias Y, Yarmush ML, Uygun K (2010). Organ reengineering through development of a transplantable recellularized liver graft using decellularized liver matrix. Nat Med.

[16] Ott HC, Clippinger B, Conrad C, Schuetz C, Pomerantseva I, Ikonomou L, Kotton D, Vacanti JP (2010). Regeneration and orthotopic transplantation of a bioartificial lung. Nat Med.

[17] Petersen TH, Calle EA, Zhao L, Lee EJ, Gui L, Raredon MB, Gavrilov K, Yi T, Zhuang ZW, Breuer C, Herzog E, Niklason LE (2010). Tissue-engineered lungs for in vivo implantation. Science.

[18] Jank BJ, Xiong L, Moser PT, Guyette JP, Ren X, Cetrulo CL, Leonard DA, Fernandez L, Fagan SP, Ott HC (2015). Engineered composite tissue as a bioartificial limb graft. Biomaterials.

[19] Goh SK, Bertera S, Olsen P, Candiello JE, Halfter W, Uechi G, Balasubramani M, Johnson SA, Sicari BM, Kollar E, Badylak SF, Banerjee I (2013). Perfusion-decellularized pancreas as a natural 3D scaffold for pancreatic tissue and whole organ engineering. Biomaterials.

[20] Burk J, Erbe I, Berner D, Kacza J, Kasper C, Pfeiffer B, Winter K, Brehm W (2013). Freeze-thaw cycles enhance decellularization of large tendons. Tissue Eng Part C Methods.

[21] Yang M, Chen CZ, Wang XN, Zhu YB, Gu YJ (2009). Favorable effects of the detergent and enzyme extraction method for preparing decellularized bovine pericardium scaffold for tissue engineered heart valves. J Biomed Mater Res B Appl Biomater.

[22] Reing JE, Brown BN, Daly KA, Freund JM, Gilbert TW, Hsiong SX, Huber A, Kullas KE, Tottey S, Wolf MT, Badylak SF (2010). The effects of processing methods upon mechanical and biologic properties of porcine dermal extracellular matrix scaffolds. Biomaterials.

[23] Quint C, Kondo Y, Manson RJ, Lawson JH, Dardik A, Niklason LE (2011). Decellularized tissue-engineered blood vessel as an arterial conduit. Proc Natl Acad Sci USA.

[24] Cebotari S, Tudorache I, Jaekel T, Hilfiker A, Dorfman S, Ternes W, Haverich A, Lichtenberg A (2010). Detergent decellularization of heart valves for tissue engineering: toxicological effects of residual detergents on human endothelial cells. Artif Organs.

[25] Faulk DM, Carruthers CA, Warner HJ, Kramer CR, Reing JE, Zhang L, D’Amore A, Badylak SF (2014). The effect of detergents on the basement membrane complex of a biologic scaffold material. Acta Biomater.

[26] Akhyari P, Aubin H, Gwanmesia P, Barth M, Hoffmann S, Huelsmann J, Preuss K, Lichtenberg A (2011). The quest for an optimized protocol for whole-heart decellularization: a comparison of three popular and a novel decellularization technique and their diverse effects on crucial extracellular matrix qualities. Tissue Eng Part C Methods.

[27] Wallis JM, Borg ZD, Daly AB, Deng B, Ballif BA, Allen GB, Jaworski DM, Weiss DJ (2011). Comparative assessment of detergent-based protocols for mouse lung de-cellularization and re-cellularization. Tissue Eng Part C Methods.

[28] Ren H, Shi X, Tao L, Xiao J, Han B, Zhang Y, Yuan X, Ding Y (2013). Evaluation of two decellularization methods in the development of a whole-organ decellularized rat liver scaffold. Liver Int.

[29] Woods T, Gratzer PF (2005). Effectiveness of three extraction techniques in the development of a decellularized bone–anterior cruciate ligament–bone graft. Biomaterials.

[30] Cartmell JS, Dunn MG (2000). Effect of chemical treatments on tendon cellularity and mechanical properties. J Biomed Mater Res.

[31] Johnson TD, Hill RC, Dzieciatkowska M, Nigam V, Behfar A, Christman KL, Hansen KC (2016). Quantification of decellularized human myocardial matrix: a comparison of six patients. Proteomics Clin Appl.

[32] Lumpkins SB, Pierre N, McFetridge PS (2008). A mechanical evaluation of three decellularization methods in the design of a xenogeneic scaffold for tissue engineering the temporomandibular joint disc. Acta Biomater.

[33] Zhou J, Fritze O, Schleicher M, Wendel HP, Schenke-Layland K, Harasztosi C, Hu S, Stock UA (2010). Impact of heart valve decellularization on 3-D ultrastructure, immunogenicity and thrombogenicity. Biomaterials.

[34] Santoso EG, Yoshida K, Hirota Y, Aizawa M, Yoshino O, Kishida A, Osuga Y, Saito S, Ushida T, Furukawa KS (2014). Application of detergents or high hydrostatic pressure as decellularization processes in uterine tissues and their subsequent effects on in vivo uterine regeneration in murine models. PLoS ONE.

[35] Li Y, Yu SM (2013). Targeting and mimicking collagens via triple helical peptide assembly. Curr Opin Chem Biol.

[36] Li Y, Foss CA, Summerfield DD, Doyle JJ, Torok CM, Dietz HC, Pomper MG, Yu SM (2012). Targeting collagen strands by photo-triggered triple-helix hybridization. Proc Natl Acad Sci USA.

[37] Li Y, Ho D, Meng H, Chan TR, An B, Yu H, Brodsky B, Jun AS, Yu MS (2013). Direct detection of collagenous proteins by fluorescently labeled collagen mimetic peptides. Bioconjugate Chem.

[38] Wainwright JM, Czajka CA, Patel UB, Freytes DO, Tobita K, Gilbert TW, Badylak SF (2009). Preparation of cardiac extracellular matrix from an intact porcine heart. Tissue Eng Part C Methods.

[39] Baptista PM, Siddiqui MM, Lozier G, Rodriguez SR, Atala A, Soker S (2011). The use of whole organ decellularization for the generation of a vascularized liver organoid. Hepatology.

[40] Soto-Gutierrez A, Zhang L, Medberry C, Fukumitsu K, Faulk D, Jiang H, Reing J, Gramignoli R, Komori J, Ross M, Nagaya M, Lagasse E, Stolz D, Strom SC, Fox IJ, Badylak SF (2011). A whole-organ regenerative medicine approach for liver replacement. Tissue Eng Part C Methods.

[41] Li Y, Mo X, Kim D, Yu SM (2011). Template-tethered collagen mimetic peptides for studying heterotrimeric triple-helical interactions. Biopolymers.

[42] San BH, Li Y, Tarbet EB, Yu SM (2016). Nanoparticle assembly and gelatin binding mediated by triple helical collagen mimetic peptide. ACS Appl Mater Interfaces.

[43] White LJ, Taylor AJ, Faulk DM, Keane TJ, Saldin LT, Reing JE, Swinehart IT, Turner NJ, Ratner BD, Badylak SF The impact of detergents on the tissue decellularization process: a ToF-SIMS study. Acta Biomater.

[44] Boudko S, Frank S, Kammerer RA, Stetefeld J, Schulthess T, Landwehr R, Lustig A, Bachinger HP, Engel J (2002). Nucleation and propagation of the collagen triple helix in single-chain and trimerized peptides: transition from third to first order kinetics. J Mol Biol.

[45] Ackerman MS, Bhate M, Shenoy N, Beck K, Ramshaw JA, Brodsky B (1999). Sequence dependence of the folding of collagen-like peptides. Single amino acids affect the rate of triple-helix nucleation. J Biol Chem.

[46] Engel J, Bächinger HP, Brinckmann J, Notbohm H, Müller PK (2005). Structure, stability and folding of the collagen triple helix. Collagen.

[47] McDonald J (2009). Handbook of Biological Statistics.

[48] Syed O, Walters NJ, Day RM, Kim HW, Knowles JC (2014). Evaluation of decellularization protocols for production of tubular small intestine submucosa scaffolds for use in oesophageal tissue engineering. Acta Biomater.

[49] He M, Callanan A (2012). Comparison of methods for whole-organ decellularization in tissue engineering of bioartificial organs. Tissue Eng Part B Rev.

[50] Hashimoto Y, Hattori S, Sasaki S, Honda T, Kimura T, Funamoto S, Kobayashi H, Kishida A (2016). Ultrastructural analysis of the decellularized cornea after interlamellar keratoplasty and microkeratome-assisted anterior lamellar keratoplasty in a rabbit model. Sci Rep.

[51] Privé GG (2007). Detergents for the stabilization and crystallization of membrane proteins. Methods.

[52] Seddon AM, Curnow P, Booth PJ (2004). Membrane proteins, lipids and detergents: not just a soap opera. Biochim Biophys Acta.

[53] Yu SM, McQuade DT, Quinn MA, Hackenberger CPR, Gellman SH, Krebs MP, Polans AS (2000). An improved tripod amphiphile for membrane protein solubilization. Protein Sci.

[54] Brown BN, Badylak SF (2014). Extracellular matrix as an inductive scaffold for functional tissue reconstruction. Transl Res.

[55] An B, Lin YS, Brodsky B (2016). Collagen interactions: drug design and delivery. Adv Drug Del Rev.

[56] Barczyk M, Carracedo S, Gullberg D (2009). Integrins. Cell Tissue Res.

[57] Hodge AJ, Highberger JH, Gottfried GJD, Schmitt FO (1960). The effects of proteases on the tropocollagen macromolecule and on its aggregation properties. Proc Natl Acad Sci USA.

[58] Drake MP, Davison PF, Bump S, Schmitt FO (1966). Action of proteolytic enzymes on tropocollagen and insoluble collagen. Biochemistry.

[59] Ryhänen L, Zaragoza EJ, Uitto J (1983). Conformational stability of type I collagen triple helix: evidence for temporary and local relaxation of the protein conformation using a proteolytic probe. Arch Biochem Biophys.

[60] Veres SP, Harrison JM, Lee JM (2014). Mechanically overloading collagen fibrils uncoils collagen molecules, placing them in a stable, denatured state. Matrix Biol.

[61] Chan TR, Stahl PJ, Li Y, Yu SM (2015). Collagen–gelatin mixtures as wound model, and substrates for VEGF-mimetic peptide binding and endothelial cell activation. Acta Biomater.

[62] Kajbafzadeh AM, Javan-Farazmand N, Monajemzadeh M, Baghayee A (2012). Determining the optimal decellularization and sterilization protocol for preparing a tissue scaffold of a human-sized liver tissue. Tissue Eng Part C Methods.

